# Aspiration pneumonia and bronchopneumonia in progressive supranuclear palsy treated with *qing fei tang*: two case reports

**DOI:** 10.1186/s13256-015-0538-3

**Published:** 2015-03-26

**Authors:** Ichiro Nozaki, Yuko Kato-Motozaki, Tokuhei Ikeda, Kazuya Takahashi, Atsuro Tagami, Chiho Ishida, Kiyonobu Komai

**Affiliations:** 1 Faculty of Medicine, Institute of Medical, Pharmaceutical and Health Sciences, Kanazawa University, 13-1, Takara-machi, Kanazawa, 920-8640 Japan; 2 Department of Neurology, National Hospital Organization Iou Hospital, Ni-73-1, Iwade-machi, Kanazawa, 920-0192 Japan; 3 Department of Internal Medicine, National Hospital Organization Iou Hospital, Ni-73-1, Iwade-machi, Kanazawa, 920-0192 Japan

**Keywords:** Aspiration pneumonia and bronchopneumonia, *Qing fei tang*, Parkinson’s syndrome, Progressive supranuclear palsy

## Abstract

**Introduction:**

*Qing fei tang*, which is used for various respiratory diseases, is useful for reducing relapse of aspiration pneumonia and bronchopneumonia in stroke, but the effect remains unknown in Parkinson's syndrome. We report two cases of Japanese patients with progressive supranuclear palsy and relapsing aspiration pneumonia and bronchopneumonia, which was successfully prevented by *qing fei tang*.

**Case presentation:**

Two Japanese men with progressive supranuclear palsy and receiving total enteral feeding (patient one (66-years-old) and patient two (76-years-old)) had experienced recurrent aspiration pneumonia and bronchopneumonia, which was unresponsive to conventional therapy. The respiratory infection developed twice at intervals of two months in patient one, and nine times at almost monthly intervals in patient two. Thereafter, they were given *qing fei tang*. After administration of *qing fei tang*, the respiratory infection reoccurred only once; after 5.5 months for patient one, and six months for patient two. Both of our patients clearly showed a reduced incidence of respiratory infection.

**Conclusions:**

Both of our patients clearly showed a reduced incidence of respiratory infection after the administration of *qing fei tang. Qing fei tang* could be useful for the prevention of recurrent aspiration pneumonia and bronchopneumonia in progressive supranuclear palsy.

## Introduction

Aspiration pneumonia and bronchopneumonia frequently occur in patients with Parkinson’s syndrome in their later stage, mainly by dysphagia. The prevention of relapsing aspiration pneumonia and bronchopneumonia is unsatisfactory in Parkinson’s syndrome.

*Qing fei tang*, which is a Chinese traditional medical mixture called *seihai-to* in Japan, has been used for the treatment of productive cough, acute and chronic bronchitis, bronchiectasis, pharyngitis, bronchial asthma, and pneumonia. A main component of *qing fei tang* is powdered extract composed of the following 16 herbs: Angelicae Radix, Ophiopogonis Tuber, Hoelen, Scutellariae Radix, Platycodi Radix, Armeniacae Semen, Gardeniae Fructus, Mori Cortex, Zizyphi Fructus, Aurantii Nobilis Pericarpium, Caulis Bambusae in Taeniis, Asparagi Radix, Fritillariae Bulbus, Glycyrrhizae Radix, Schisandrae Fructus, and Zingiberis Siccatum Rhizoma [1]. It is reported that *qing fei tang* is effective for reducing the relapse rate of aspiration pneumonia and bronchopneumonia in patients with stroke or recurrent laryngeal nerve palsy [1]. To the best of our knowledge, there have been no reported cases of Parkinson’s syndrome with recurrent aspiration pneumonia successfully treated by *qing fei tang*.

In progressive supranuclear palsy (PSP), aspiration pneumonia and bronchopneumonia frequently occurs, and often is refractory. We considered whether *qing fei tang* would be effective for the prevention of relapsing aspiration pneumonia and bronchopneumonia in PSP as well as stroke. If so, then *qing fei tang* could be a new treatment for recurrent and refractory respiratory infection in PSP.

We present two cases of Japanese patients with PSP and relapsing aspiration pneumonia and bronchopneumonia despite receiving total enteral feeding and conventional medication. We describe the successful treatment of their recurrent respiratory infections by *qing fei tang*.

## Case presentation

### Patient one

**Figure 1:**
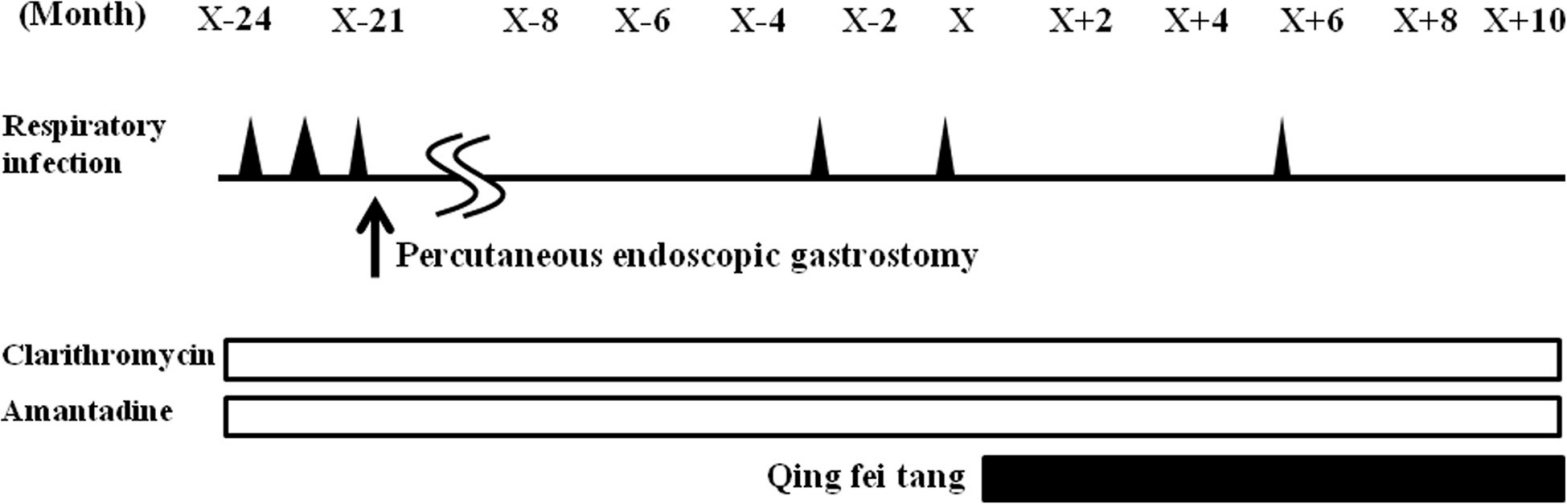
**The clinical course of patient one.** The incidence of aspiration pneumonia and bronchopneumonia, which are shown by black triangles was exhibited before and after administration of *qing fei tang* at X month in patient one.

A 63-year-old Japanese man presented with speech difficulty, standing instability, drinking difficulty, and slow movement at first. At 12 months after his initial presentation, his neurological examination revealed cognitive decline, masked face, vertical oculomotor disturbance, dysarthria, bradykinesia, trunk-dominant muscular rigidity, and a tendency to fall. Magnetic resonance imaging of his head showed mild atrophy of the midbrain tegmentum. Probable PSP was diagnosed according to the National Institute of Neurological Disorders and Stroke and the Society for PSP (NINDS-SPSP) diagnostic criteria [2]. At 10 months after the diagnosis, he was confined to a wheelchair, and aspiration pneumonia occurred three times at monthly intervals. Although a percutaneous endoscopic gastrostomy (PEG) was performed immediately after his last pneumonia, pneumonia reoccurred twice at 18 and 20 months after his PEG regardless of treatment with clarithromycin and amantadine (Figure 1). A daily dose of 9g of *qing fei tang* (Tsumura & Co., Tokyo, Japan) was started, and aspiration pneumonia reoccurred only once, at 5.5 months after the start of treatment (Figure 1). The bacteria in his sputum were the same as before starting *qing fei tang* (*Pseudomonas aeruginosa* and methicillin-resistant *Staphylococcus aureus* (MRSA)), except for *Streptococcus agalactiae*.

### Patient two

**Figure 2:**
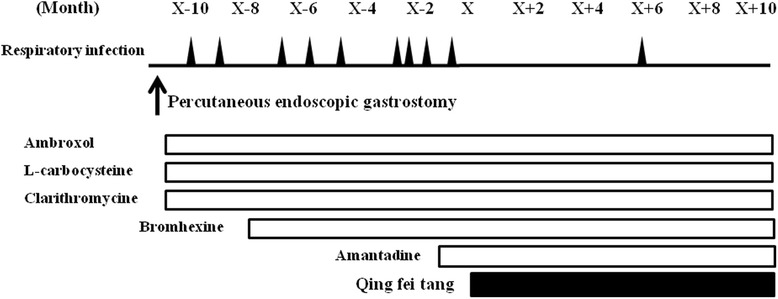
**The clinical course of patient two.** The incidence of aspiration pneumonia and bronchopneumonia, which are shown by black triangles was shown before and after the administration of *qing fei tang* at X month in patient two.

A 74-year-old Japanese man presented to us with a three-year history of difficulty in moving his upper limbs, a tendency to fall, and slow movements. His neurological examination revealed supranuclear vertical gaze palsy, dysarthria, bradykinesia, muscular rigidity, and loss of postural reflex. Magnetic resonance imaging of his head demonstrated severe atrophy of his midbrain tegmentum. These clinical manifestations led to a diagnosis of probable PSP according to NINDS-SPSP criteria [2]. A PEG was performed in a bedridden condition because of progressive dysphagia 23 months after the diagnosis, but then aspiration pneumonia and bronchopneumonia developed nine times during 10 months, at intervals of about one month, with medication including ambroxol, L-carbocysteine, clarithromycin, bromhexine, and amantadine (Figure 2). *Qing fei tang* was given daily at a dose of 9g, and then aspiration pneumonia occurred only once, six months after starting the treatment (Figure 2). The types of bacteria in his sputum did not change compared with before starting *qing fei tang* (*P. aeruginosa*, MRSA and *Klebsiella pneumoniae*).

## Discussion

We present two cases of Japanese patients with PSP who experienced recurrent aspiration pneumonia and bronchopneumonia regardless of receipt of total enteral feeding and conventional therapy. Patient one experienced recurrence twice at intervals of two months and patient two experienced recurrence nine times at almost monthly intervals. The respiratory infection developed only once, at 5.5 months (patient one) and six months (patient two) after the administration of *qing fei tang*.

After administration of *qing fei tang*, the incidence of aspiration pneumonia and bronchopneumonia fell in both of our patients, and the interval until onset of respiratory infection was prolonged. No adverse effects induced by *qing fei tang* were found in either of our patients.

Aspiration pneumonia and bronchopneumonia is the leading cause of death in patients with Parkinson’s syndrome, including dementia with diffuse Lewy bodies, corticobasal degeneration, PSP, and multiple system atrophy [3]. Onset of dysphagia deteriorates the prognosis of Parkinson’s syndrome [3], and may cause subsequent silent aspiration [4]. Good control of recurrent aspiration pneumonia and bronchopneumonia is important to improve the prognosis in Parkinson’s syndrome. To date, conventional treatment including clarithromycin and amantadine has been unsatisfactory. Furthermore, total enteral feeding is not completely safe because aspiration pneumonia can develop due to silent aspiration, even if patients do not feed orally. In our patient one, although no aspiration pneumonia occurred after PEG for a while, afterwards respiratory infection reoccurred twice in a relatively short period. It has been reported that *qing fei tang* was effective for relapsing aspiration pneumonia in seven patients with stroke treated with *qing fei tang* including four patients without oral intake, compared with eight patients with stroke treated with conventional therapy including six patients without oral intake [1]. Both of our patients were without oral intake, and we also found that *qing fei tang* could reduce relapse of aspiration pneumonia and bronchopneumonia.

The preventive effects of *qing fei tang* against aspiration pneumonia are considered to occur by a mechanism other than the improvement of dysphagia, because the swallowing reflex could not be improved by *qing fei tang* [1]. In a previous report, xanthine oxidase activity was elevated in the lung tissues of aspiration pneumonia-model mice, but the activation was markedly inhibited by *qing fei tang* [5]. Pretreatment with *qing fei tang* is considered to reduce oxygen radicals produced by inflammation in the lungs, and to reduce the mortality rate of mice with aspiration pneumonia [5]. In our two patients, while *qing fei tang* could inhibit the respiratory infection effectively, the types of bacteria detected in their sputum were almost the same both before and after administration of *qing fei tang*. The effects of *qing fei tang* could not be explained by the change in the types of bacteria, but could partially be explained by the above-mentioned defense mechanism, namely, the reduction of oxygen radicals.

In our case series, it was a new finding that administration of *qing fei tang* could lead to a reduction in the incidence of aspiration pneumonia and bronchopneumonia, and prolong the interval until the onset of respiratory infection in PSP. *Qing fei tang* may be a safe available option for preventive therapy of recurrent aspiration pneumonia and bronchopneumonia by a different mechanism from conventional medication. The reduction of respiratory infection may prolong the survival time in PSP. *Qing fei tang* should be considered when aspiration pneumonia cannot be kept under control in patients with PSP using conventional therapy and total enteral feeding. Further large-scale, ideally mechanistic, studies may better clarify whether *qing fei tang* can reduce the relapse rate of aspiration pneumonia in patients with PSP, compared to a control.

## Conclusions

We report two cases of patients with PSP and recurrent aspiration pneumonia and bronchopneumonia, the latter of which was successfully treated by *qing fei tang. Qing fei tang* could prevent recurrent aspiration pneumonia and bronchopneumonia in patients with PSP, and may be an option for treatment in addition to conventional therapy.

## Consent

Written informed consent was obtained from the patients for publication of this case series. The copies of the written consent are available for review by the Editor-in-Chief of this journal.

## Abbreviations

MRSA: Methicillin-resistant *Staphylococcus aureus*
NINDS-SPSP: National Institute of Neurological Disorders and the Society for PSP diagnostic criteria
PEG: Percutaneous endoscopic gastrostomy
PSP: Progressive supranuclear palsy
